# Specific Gain and Loss of Co-Expression Modules in Long-Lived Individuals Indicate a Role of circRNAs in Human Longevity

**DOI:** 10.3390/genes13050749

**Published:** 2022-04-24

**Authors:** Ming-Xia Ge, Jian-Jun Jiang, Li-Qin Yang, Xing-Li Yang, Yong-Han He, Gong-Hua Li, Qing-Peng Kong

**Affiliations:** 1 State Key Laboratory of Genetic Resources and Evolution/Key Laboratory of Healthy Aging Research of Yunnan Province, Kunming Institute of Zoology, Chinese Academy of Sciences, Kunming 650223, China; gemingxia@mail.kiz.ac.cn (M.-X.G.); jianjunjiang@outlook.com (J.-J.J.); yangliqin@mail.kiz.ac.cn (L.-Q.Y.); yang1992xingli@163.com (X.-L.Y.); heyonghan-2008@163.com (Y.-H.H.); ligonghua@mail.kiz.ac.cn (G.-H.L.); 2 CAS Center for Excellence in Animal Evolution and Genetics, Chinese Academy of Sciences, Kunming 650223, China; 3 Kunming Key Laboratory of Healthy Aging Study, Kunming 650223, China; 4 KIZ/CUHK Joint Laboratory of Bioresources and Molecular Research in Common Diseases, Kunming 650223, China; 5 Kunming College of Life Science, University of Chinese Academy of Sciences, Beijing 100049, China

**Keywords:** long-lived families, circular RNA, co-expression, healthy aging

## Abstract

Deep RNA sequencing of 164 blood samples collected from long-lived families was performed to investigate the expression patterns of circular RNAs (circRNAs). Unlike that observed in previous studies, circRNA expression in long-lived elderly individuals (98.3 ± 3.4 year) did not exhibit an age-accumulating pattern. Based on weighted circRNA co-expression network analysis, we found that longevous elders specifically gained eight but lost seven conserved circRNA-circRNA co-expression modules (c-CCMs) compared with normal elder controls (spouses of offspring of long-lived individuals, age = 59.3 ± 5.8 year). Further analysis showed that these modules were associated with healthy aging-related pathways. These results together suggest an important role of circRNAs in regulating human lifespan extension.

## 1. Introduction

Aging has been considered as the largest risk factor in the process of various human diseases such as cardiovascular abnormalities, neurological dysfunctions, immunological disorders and cancers [1,2,3,4]. The intervention of aging and a better understanding of healthy aging mechanisms are in severely urgent needs to be explored. Evidence from both transcriptomic and epigenetic perspectives has shown that long-lived individuals possess the capability of delaying age-related diseases, thus regarded as good models for studying healthy aging regulations [5,6,7].

Recent studies suggested that noncoding RNAs are involved in healthy aging and/or age-related diseases [8,9,10]. It remains, however, largely unknown whether circular RNAs (circRNAs), a class of endogenous noncoding RNA with a covalently closed continuous loop predominantly generated from back-spliced exons [11], and acting as ‘microRNA sponges’ or ‘scaffolding’ for RNA-binding protein [12,13], in human longevity. Increasing evidence has revealed the crucial roles of circRNAs in multiple biological processes and even in human diseases [14,15]. For instance, several circRNAs were related with age-related diseases, including neurodegenerative diseases [16], cardiovascular diseases [17], type 2 diabetes [18] and, even, cancers [19]. Nevertheless, their roles in the process of human lifespan extension are largely unexplored. In this study, we investigated the circRNAs expression pattern of longevous families, from a Chinese cohort of longevity. We found that the most significant signature in long lived people is the gain and loss of circRNAs co-expression modules across their healthy lifespan. These modules have been further proved to be related to healthy aging-related pathways, suggesting that they may most likely play a role in regulating healthy aging.

## 2. Materials and Methods

### 2.1. Sampling and RNA-seq

Human peripheral blood samples of 164 individuals from longevous families were collected from Hainan Province in southern China in two batches, 50 samples [5] and 114 samples, respectively. The 164 biospecimens included 71 long-lived individuals (LLI, age: 98.3 ± 3.4 year), 57 offspring of LLIs (F1, age: 60.9 ± 6.8 year) and 36 spouses of F1 (F1SP, age: 59.3 ± 5.8 year). White blood cells were isolated from the peripheral blood using red blood cell lysis buffer (Tiangen, Beijing, China) and centrifugation at 4000 rpm for 10 min at room temperature. Total RNA samples were extracted using the TRIzol method. The rRNA-depleted RNA-seq libraries were prepared following the instructions contained in the Ribo-Zero kit (China) and were deeply sequenced using the Illumina HiSeq 4000 platform. All research protocols were approved by the Ethics Committee at the Kunming Institute of Zoology, Chinese Academy of Sciences (Approval Code: SMKX-20141220-74; Approval Date: 20 December 2014).

### 2.2. CircRNA Prediction and Quantification

The raw total RNA-seq data were first trimmed and cleaned by SolexaQA [20] using default parameters. The clean data were then aligned to the human reference genome (hg19) using TopHat2 [21]. The CIRCexplorer pipeline [22] was used to identify circRNA transcripts following the standard tutorial. After applying the filtering criteria (junction reads ≥ 2 and expression in at least 5% of samples) adopted previously [23,24], circRNAs with high confidence were retained. To evaluate the expression levels of circRNAs in different samples, back-spliced junction reads of circRNAs were normalized to reads per million mapped reads (RPM) [22]. Linear RNA reads were quantified and scaled using FPKM by Cufflinks [25]. Batch effects of all samples was estimated and visualized by principal component analysis (PCA) using the FactoMineR and factoextra R packages. The ‘removeBatchEffect’ function in the ‘limma’ package [26] was used to remove batch effects in the R platform. As a result, a total of 17 081 high-quality circRNAs (Table S2) were identified.

### 2.3. Analysis of Cell-Type Composition and General circRNA Expression Pattern

We used average linkage hierarchical clustering to cluster and label samples in the dendrogram plot, which provides information on how objects are iteratively merged together. The clustering height is the value of the criterion associated with the clustering method for the particular agglomeration [27]. We set a cutoff value of 13 to detect and remove outlier samples based on the clustering tree (Figure S1). As a result, four outlier samples were removed (X457, X459, X388 and X611), and 160 RNA-seq profiles were retained for subsequent analysis.

To evaluate any potential bias of cellular components on circRNA analysis, we performed Pearson correlation analysis between age and cellular components of blood samples (including LLIs, F1SPs and F1s). No significant associations between neutrophil, lymphocyte cell ratios and age were observed (*p* = 0.5193 and 0.9082, respectively) (Figure S2) (Figure S2). The RPM values were visualized using the ggplot2 package in R. The density plot was visualized by kernel smoothing using the kernel density estimate. Statistical significance among different groups was evaluated by ‘Two-way ANOVA’ (Design: RPM ~ group + gender).

### 2.4. Construction of circRNA-circRNA Co-Expression Network and Module Preservation Analysis

CircRNA-circRNA co-expression network analysis was performed using the ‘WGCNA’ package [28], with a minimum circRNA cutoff of 100. The Module Preservation tool in ‘WGCNA’ was used to calculate the preservation of each module. The Zsummary value was used to assess conservation, with a value of 10 regarded as strong preservation [29].

The co-expression network plots were constructed using Cytoscape (v3.7.1) [30] after removing weak associations (weight value < 0.1). Clustering coefficient and network density were calculated using the Network Analyzer program in Cytoscape [30]. Statistical significance was ascertained using the *t*-test function in R language.

### 2.5. CircRNA-Gene (mRNA) Co-Expression Analysis and Gene Enrichment Analysis

For circRNAs in each c-CCM, Pearson correlation coefficients (r) in the R package were used to determine correlations with mRNA. The FPKM (fragments per kilobase of transcript per million fragments) values of annotated genes acquired from the human genome (hg19) were subjected to co-expression analysis. Genes with FPKM < 1 were filtered out and 10,260 genes were ultimately used for co-expression analysis. Thresholds (Pearson r ≥ 0.75 and *p* < 0.05) were employed to obtain genes strongly co-expressed with circRNAs.

The strongly co-expressed genes in each c-CCM were then subjected to Gene Ontology (GO) and KEGG pathway enrichment analysis using the ‘clusterprofiler’ package [31] and visualized using the ‘ggplot2’ package in the R platform.

## 3. Results

### 3.1. Identification and General Expression Pattern of circRNAs in Longevous Families

**Figure 1:**
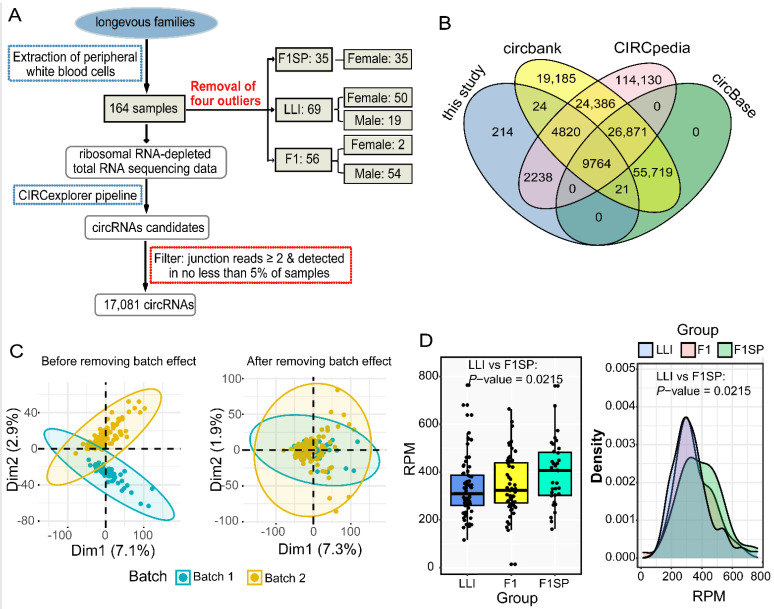
Identification and general expression pattern of circRNAs in longevous families. (**A**) CircRNA identification pipeline used in this study. CIRCexplorer pipeline [17] was used for rRNA-depleted RNA-seq data. In total, 17,081 high-quality circRNA transcripts were identified for next-step analysis. (**B**) Comparison between annotated circRNAs acquired from three databases (circBase [33], circbank [32] and CIRCpedia [34]) and circRNAs identified in this study. (**C**) The PCA plot was used to estimate batch effect of samples. (**left panel**: distribution of samples before removing batch effect; **right panel**: distribution of samples after removing batch effect). (**D**) CircRNA expression levels in LLI, F1SP-F and F1 groups (**left panel**: RPM distribution; **right panel**: kernel density histogram).

For a better understanding of longevity regulation from the perspective of circRNAs, we performed deep RNA sequencing (RNA-seq) on peripheral blood samples collected from 164 Chinese individuals in longevous families, including 71 long-lived individuals (LLIs, age = 98.3 ± 3.4 year), 57 offspring of LLIs (F1, age = 60.9 ± 6.8 year) and 36 spouses of F1 (F1SP, age = 59.3 ± 5.8 year) (Figure 1A and Table S1). We identified 17,081 circRNAs with high confidence (Figure 1B and Table S2), 98.75% (16,867/17,081) of which were reported in three public circRNA databases (circbank [32], circBase [33] and CIRCpedia [34]).

To investigate the expression patterns of the circRNAs identified in our long-lived family samples, batch effect was removed and visualized by principal component analysis (PCA) (Figure 1C). Subsequently, we employed outlier detection and cell-type composition analysis to remove biospecimens using the R platform hclust function with a cutoff value of 13. In total, 160 samples were retained for further analysis (Figures S1 and S2). We first examined the differences in circRNA expression between the longevity subjects and younger adult controls, and found that the average expression level of circRNAs in the LLI group was significantly lower than that in normal elders (F1SP group) after gender bias adjustment (see methods; Figure 1D). In addition, as shown in the kernel density histogram of circRNAs, both the LLI and F1 groups, but not the F1SP group, displayed similar distributions based on smoothed density estimation analysis (Figure 1D).

### 3.2. Specific circRNAs Co-Expression Modules Were Observed Gained or Lost in Long-Lived Individuals

**Figure 2:**
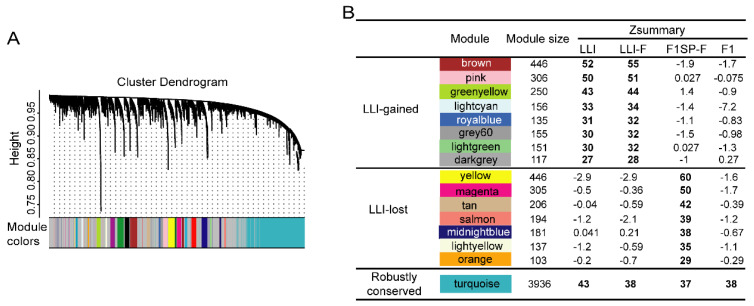
Construction of weighted circRNA co-expression network. (**A**) Module tree plot of circRNA-circRNA in all samples generated by blockwiseModules and plotDendroAndColors parameters in WGCNA package. Each color represents one co-expression module. (**B**) Module preservation analysis results. Each color represents one co-expression module. Preservation degree of modules was assessed by Zsummary scores (value > 10 was regarded as strong preservation). One module with higher Zsummary score indicates its stronger conservation in the sample group [29].

**Figure 3:**
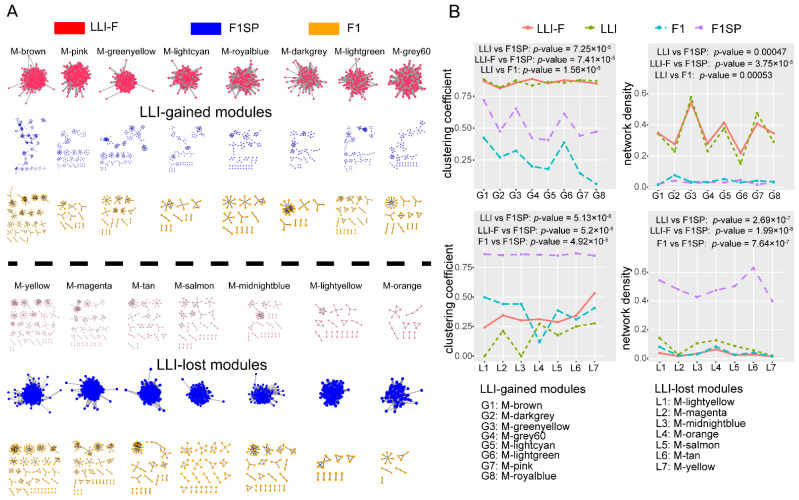
Specific circRNAs co-expression modules were observed gained or lost in long-lived individuals. (**A**) Clustering map of 15 c-CCMs. Upper panel: eight LLI-gained c-CCMs were scattered in F1 and F1SP samples, but clearly clustered in LLI samples; Lower panel: seven LLI-lost c-CCMs exhibited similar discrete clustering distribution in LLI and F1 and were clustered into modules in F1SP. (**B**) Network density and clustering coefficient analysis of LLI-gained and LLI-lost modules in LLI, F1 and F1SP.

To explore the network characters of circRNAs in LLIs, we built a broad weighted circRNA-circRNA co-expression network using weighted gene co-expression network analysis (WGCNA) [28], and identified 25 circRNA-circRNA co-expression modules (CCMs) in our samples (Figure 2A and Table S3). Module-preservation analysis [29] showed that 16 out of 25 CCMs were conserved (Zsummary > 10) (Figure 2B and Figure S3). One module, which included 3936 circRNAs (Figure 2B), had similar Zsummary values among four groups, i.e., LLI, female LLI (LLI-F), female F1SP (F1SP-F) and F1. However, eight out of 16 modules showed specifically high conservation in the LLI and LLI-F groups but disappeared in the F1SP and F1 groups (Figure 2B and Figure 3A). In contrast, the remaining seven modules were conserved in the F1SP group but were not clustered in any of the other three groups (Figure 2B and Figure 3A). These results suggest that the LLI (including LLI-F) group gained eight but lost seven conserved CCMs (c-CCMs) compared with the normal elderly controls (F1SP). 

Network density and clustering coefficient analysis revealed that the eight LLI-gained c-CCMs had significantly higher clustering coefficients in LLI (LLI-F) as well as higher network densities than in F1SP and F1 (Figure 3B and Table S3). In contrast, the seven LLI-lost c-CCMs showed significantly lower clustering efficiencies and network densities in LLI and F1 than in F1SP (Figure 3B), suggesting that the gained modules were specific to both the LLI and LLI-F groups, but the lost modules were non-detected in the LLI, LLI-F and F1 groups. To filter out the effect of aging on the circRNAs of these modules, age-circRNA expression association analyses were conducted in the middle-aged samples (age: 45–86year old; including all F1 and F1SP subjects) as previously described [5]. The results showed that only 0.78% (56/7224) circRNAs displayed associations between their expression and age, with *p*-value < 0.01 (Table S4).

### 3.3. LLI-Gained/Lost c-CCMs Were Proved to Be Related to Healthy Aging-Related Pathways

**Figure 4:**
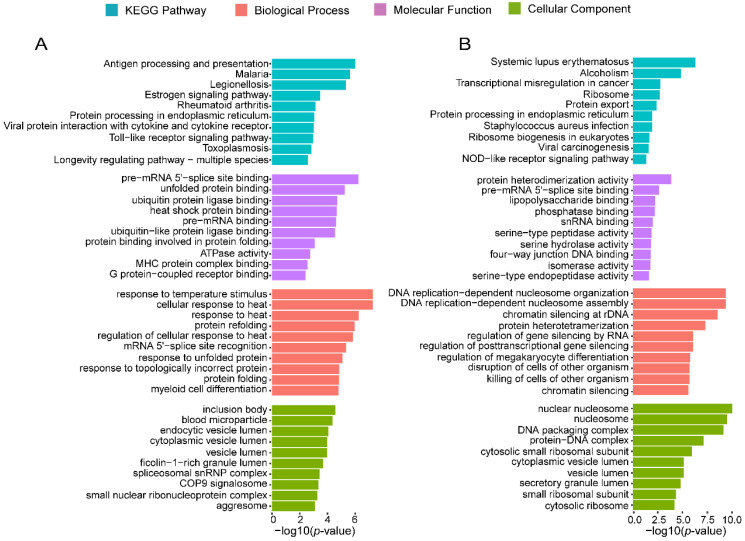
Functional enrichment of c-CCMs-related genes. (**A**,**B**) Functional classification of genes strongly (r > 0.75) co-expressed with LLI-gained and -lost c-CCMs. Top 10 KEGG pathways are shown. (**A**) LLI-gained c-CCMs. (**B**) LLI-lost c-CCMs.

To address why some c-CCMs were gained and some were lost in the LLI group, we explored their potential functions by performing circRNA-gene (mRNA) co-expression network analysis based on Pearson correlation. We found 553 circRNAs in the gained c-CCMs that were strongly co-expressed (Pearson’s r > 0.75) with 100 genes and 952 circRNAs in the lost c-CCMs that were closely correlated (Pearson’s r > 0.75) with 125 genes (Table S5). KEGG analysis showed that the 100 genes co-expressed with LLI-gained c-CCMs were markedly enriched in lifespan extension-related pathways, such as longevity regulating pathway-multiple species (hsa04213) and toll-like receptor signaling pathway (hsa04620) (Figure 4A). On the other hand, the 125 LLI-lost c-CCM-related genes were clustered into ribosome, tumorigenesis and immune system-associated pathways, including ribosome (hsa03010), ribosome biogenesis (hsa03008), viral carcinogenesis (hsa05203), transcriptional misregulation in cancer (hsa05202), IL-17 signaling pathway (hsa04657) and NOD-like receptor signaling pathway (hsa04621) (Figure 4B and Table S6), indicating that at least some of the circRNAs in the lost c-CCMs may be related to translation regulation and immune response. Interestingly, both the gained and lost module-related genes were enriched in infectious disease-related pathways, including malaria (hsa05144), legionellosis (hsa05134), toxoplasmosis (hsa05145) and *Staphylococcus aureus* infection (hsa05150).

The above results indicated that the LLI-gained/lost c-CCMs were significantly associated with several functionally important pathways, which may participate in the healthy aging process. Indeed, previous studies have suggested that most of the significantly enriched pathways identified in our results are associated with healthy aging. For instance, the ribosome biogenesis process has been causally linked to longevity and healthy aging [35,36,37], and prior studies have emphasized the importance of the toll-like receptor signaling process [38,39] and IL-17 signaling [40] in healthy life span.

## 4. Discussion

Here, by generating and analyzing deep RNA sequencing data of 164 blood samples collected from the long-lived families, we conducted a genome-wide investigation of circRNA expression patterns in a long-lived Chinese cohort for the first time. Different from the previous observations that circRNAs appear to accumulate with age in the neural tissues of mice [41], Drosophila [42] and Caenorhabditis elegans [43], we found that the overall circRNA expression in the LLIs was significantly lower than that in the younger controls (viz., F1SPs). As circRNAs are expressed in a tissue-specific manner [14] and not easily degraded by nucleases owing to the special loop structure [44], our result indicated that there may be some potential circRNA degradation or generation mechanism in peripheral blood of the longevous individuals.

Importantly, our study discovered 15 circRNA-circRNA co-expression modules that were specifically gained or lost in the longevous individuals. Previous researches have reported the regulatory role of circRNAs in certain biological process [12,13,45,46], our study showed that both the gained and lost module-related genes were enriched in infectious disease-related pathways. This suggests that these elders might have a history of infection, which could be related to life in the early and middle twentieth century when medical health care was poorer and contagious diseases were prevalent. It seems that these circRNAs may be associated with previous responses to infectious diseases.

Given that these modules, as predicted by mRNA-circRNA co-expression analysis, were closely related to processes involved in lifespan extension, the gain and loss of these c-CCMs in very long-lived individuals are unlikely to be a random process but rather contribute to healthy human aging and may represent a new target for the regulation of healthy human aging. Our findings thus provide a basis for studies on circRNAs and healthy aging. Future functional insights into the mechanisms underlying life extension could be of great help in developing new avenues for anti-aging.

## Data Availability

The RNA-seq data supporting the current study have not been deposited in a public repository due to the regulations of the China Human Genetic Resources Administration Office but are available from the corresponding author upon request. This study did not generate new code.

## Supplementary Materials

The following supporting information can be downloaded at: https://www.mdpi.com/article/10.3390/genes13050749/s1, Figure S1: Outlier detection in 164 samples; Figure S2: Pearson correlation analysis between age and cellular components of blood samples; Figure S3: Module preservation analysis; Table S1: Information and biological parameters (gender, age etc) of the participants enrolled in the study; Table S2: Full lists of detected circRNAs with high confidence in our study (n = 17,081); Table S3: Detailed information of circRNA co-expression modules obtained by WGCNA; Table S4: Results of Pearson correlation analysis between age and circRNA expression in the middle-aged samples; Table S5: List and details of genes co-expressed with LLI-gained/lost modules; Table S6: Gene Ontology and KEGG Pathway enrichment results of target modules-related genes.
