# Supplementary material for: Specific Gain and Loss of Co-Expression Modules in Long-Lived Individuals Indicate a Role of circRNAs in Human Longevity
Source: Genes (Basel). 2022 Apr 24;13(5):749. doi: 10.3390/genes13050749 (PMC9140997; doi:10.3390/genes13050749)
Supplement: Supplementary file 1 [file genes-13-00749-s001.zip › Supplementary data.pdf]

Supplementary data

Figure S1. Outlier detection in 164 samples. Four outliers (samples above red line) were removed with a cut-off value of 13 by hierarchical clustering.

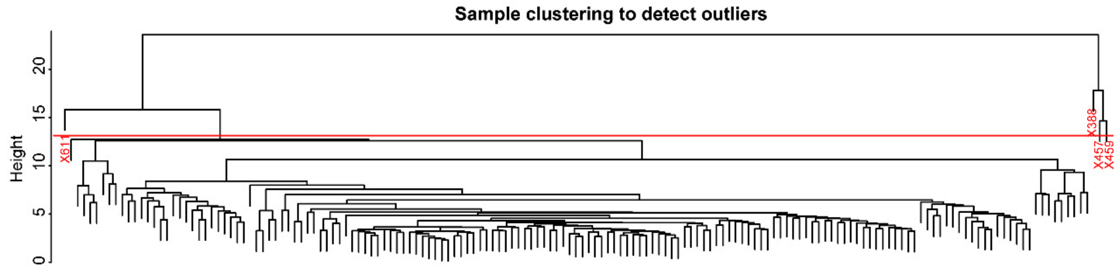

Figure S2. Pearson correlation analysis between age and cellular components of blood samples. No significant associations between neutrophil, lymphocyte cell ratios and age were observed (p = 0.5193 and 0.9082, respectively).

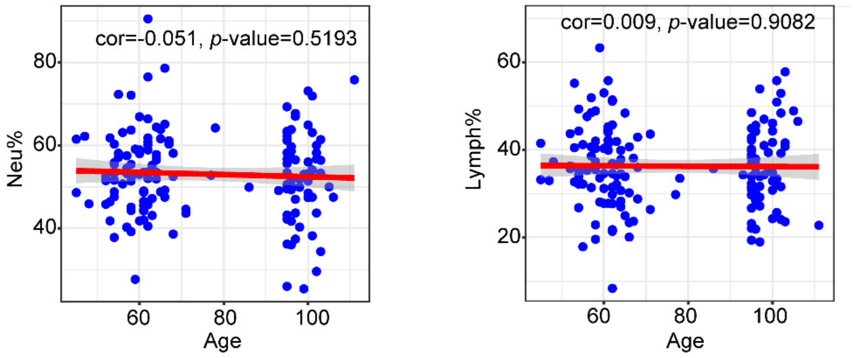

Figure S3. Module preservation analysis. Preservation degree of modules was

assessed by ‘Zsummary’ (value > 10 was regarded as strong preservation (above green line)).

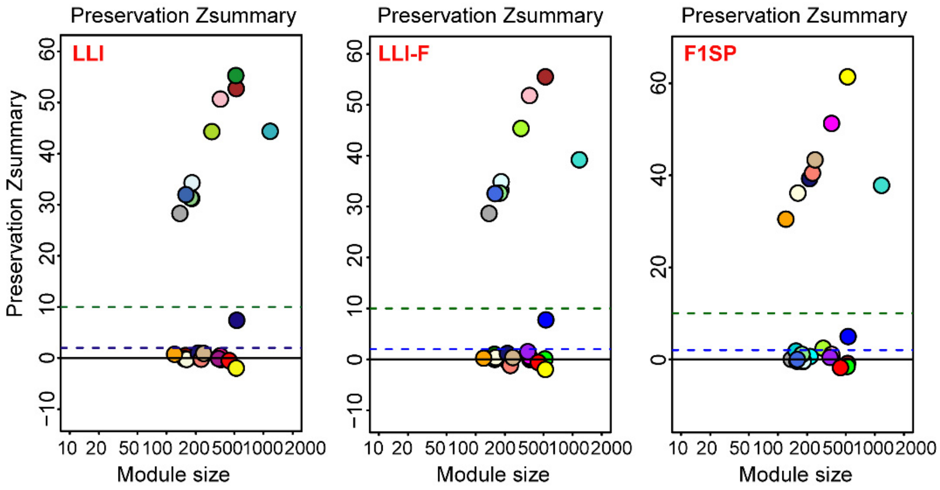

Table S1. Information and biological parameters (gender, age etc) of the participants enrolled in the study.

Table S2. Full lists of detected circRNAs with high confidence in our study (n=17081).

detailed analysis result of co-expressed circRNAs-genes in LLI-gained/lost modules.

Table S3. Detailed information of circRNA co-expression modules obtained by WGCNA.

Table S4. Results of Pearson correlation analysis between age and circRNA expression in the middle-aged samples.

Table S5. List and details of genes co-expressed with LLI-gained/lost modules.

Table S6. Gene Ontology and KEGG Pathway enrichment results of target modules-related genes. Filtering based on p-value with a cutff of 0.05.
